# Association of long noncoding RNAs expression levels and their gene polymorphisms with systemic lupus erythematosus

**DOI:** 10.1038/s41598-017-15156-4

**Published:** 2017-11-09

**Authors:** Jun Li, Guo-Cui Wu, Tian-Ping Zhang, Xiao-Ke Yang, Shuang-Shuang Chen, Lian-Ju Li, Shu-Zhen Xu, Tian-Tian Lv, Rui-Xue Leng, Hai-Feng Pan, Dong-Qing Ye

**Affiliations:** 1 Department of Epidemiology and Biostatistics, School of Public Health, Anhui Medical University, Anhui, P. R. China; 2 Anhui Province Key Laboratory of Major Autoimmune Diseases, Anhui, P. R. China; 3 Jiangyin Center for Disease Control and Prevention, Jiangsu, P. R. China

## Abstract

Increasing evidence has demonstrated the association between long noncoding RNAs (lncRNAs) and multiple autoimmune diseases. To explore four lncRNAs (GAS5, lnc-DC, linc0597 and linc0949) expression levels and gene polymorphisms in systemic lupus erythematosus (SLE), a two stage design was applied. In the first stage, 85 SLE patients and 71 healthy controls were enrolled to investigate the lncRNAs expression levels. Then, 1260 SLE patients and 1231 healthy controls were included to detect the single nucleotide polymorphisms (SNPs) in the differentially expressed lncRNAs identified in the first stage. Linc0597, lnc-DC and GAS5 expression levels were significantly lower in SLE patients than healthy controls (*P* < 0.001, *P* < 0.001, *P* = 0.003 respectively). Association of five SNPs (rs10515177, rs2070107, rs2632516, rs2877877, rs2067079) with SLE risk were analyzed. No significant association was observed between these gene polymorphisms and susceptibility to SLE (all *P* > 0.010), and we did not find significant association between any genotypes at five SNPs and their respective lncRNAs expression in SLE (all *P* > 0.010). In summary, the expression levels of linc0597, lnc-DC and GAS5 are decreased in SLE patients, but their gene polymorphisms are not associated with SLE risk, and do not influence their expression levels.

## Introduction

Systemic lupus erythematosus (SLE) is a chronic multisystem autoimmune disease which is characterized by multiple autoantibody production, formation of immune complexes that result in multiple tissue or organ damages^1–3^. It has been revealed that dysregulation of the immune system, including abnormal T-cell, B-cell, and dendritic cells (DCs) responses, participates in the pathogenesis of SLE^4–6^. However, to date, the exact pathogenic mechanism of SLE is still unknown. Over the past decades, experimental and clinical studies indicated that the interaction of genetic, epigenetic, environmental, hormonal, and immunoregulatory factors may be involved in the initiation and promotion of SLE^1,7^. Many genes associated with susceptibility to SLE have been identified through the genome-wide association studies (GWAS).

More than 80% of the human genome is transcribed into RNA transcripts with little or no protein-coding capability^8^. Besides many widely studied classes of short noncoding RNA (ncRNA), such as microRNAs (miRNA), long noncoding RNA (lncRNA) is a class of ncRNA longer than 200 nucleotides, which have emerged as important regulators of diverse biological functions^9^. Although the accurate functions of lncRNAs remains largely unclear, a number of studies have revealed that lncRNAs participate in various critical biological processes, such as chromatin remodeling, gene transcription, RNA splicing, and protein transport diverse mechanisms^10–12^, implicating their role in a wide range of complex human diseases^13,14^.

Recently, a number of lncRNAs have been reported to be involved in the pathogenesis of immune-mediated inflammatory diseases^15^, such as rheumatoid arthritis (RA)^16,17^, autoimmune thyroid disease^18^ and SLE^19,20^. Growth arrest specific 5 (GAS5), a kind of lncRNA, has been linked with increased susceptibility of SLE in a murine model^21^. Moreover, 1q25, the chromosomal locus of GAS5, has been shown to be related with human SLE development in genetic studies^22,23^. Wu *et al*.^20^ reported that linc0597 were significantly decreased in patients with SLE, and linc0949 may be a potential biomarker for diagnosis, disease activity and therapeutic response in SLE. Wang *et al*.^24^ identified a kind of lncRNA, lnc-DC, which was exclusively expressed in human conventional DCs, and regulated DCs differentiation to stimulate T cell activation.

Based on the available evidence and our recent study on the plasma expression of lncRNAs^25^, we hypothesized that GAS5, lnc-DC, linc0597 (BZRAP1-AS1) and linc0949 (OIP5-AS1) may play a critical role in the pathogenesis of SLE. In the present study, we aimed to investigate the expression levels of these lncRNAs in peripheral blood mononuclear cells (PBMCs) from SLE patients and healthy controls, as well as the association of their gene polymorphisms with susceptibility to SLE and their expression levels.

## Results

### Characteristics of study subjects

The demographic characteristics, clinical manifestations, laboratory measurements and main medical therapy of the 85 SLE patients and 71 healthy controls in stage one are summarized in Table S1. The basic characteristics of SLE patients and healthy controls in stage two (phase I, phase II and pooled result) are presented in Tables S2–S4.

### Expression levels of lncRNAs in SLE patients

**Figure 1:**
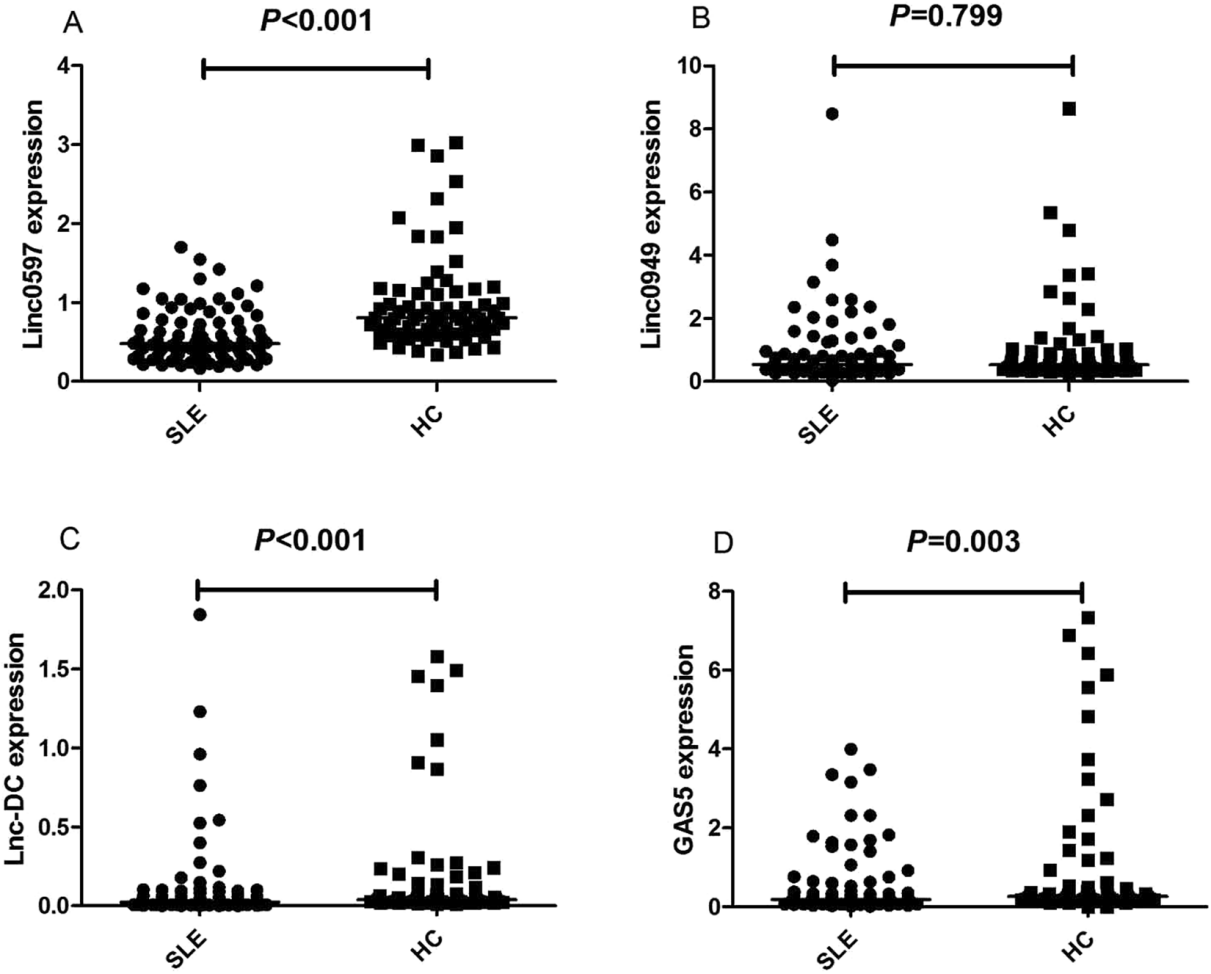
Comparison of expression of lncRNAs between different groups. Each symbol represents an individual subjects; horizontal lines indicate median values. The expression levels of the lncRNAs in 85 SLE patients, 71 healthy controls were analyzed by qRT-PCR and normalized by β-actin. (**A**) Decreased expression of linc0597 in patients with SLE versus healthy controls. (**B**) The expression of linc0949 in SLE and healthy controls did not show any difference. (**C**) The expression of lnc-DC in SLE was significantly lower than healthy controls. (**D**) The expression of GAS5 in SLE was significantly lower than healthy controls.

**Figure 2:**
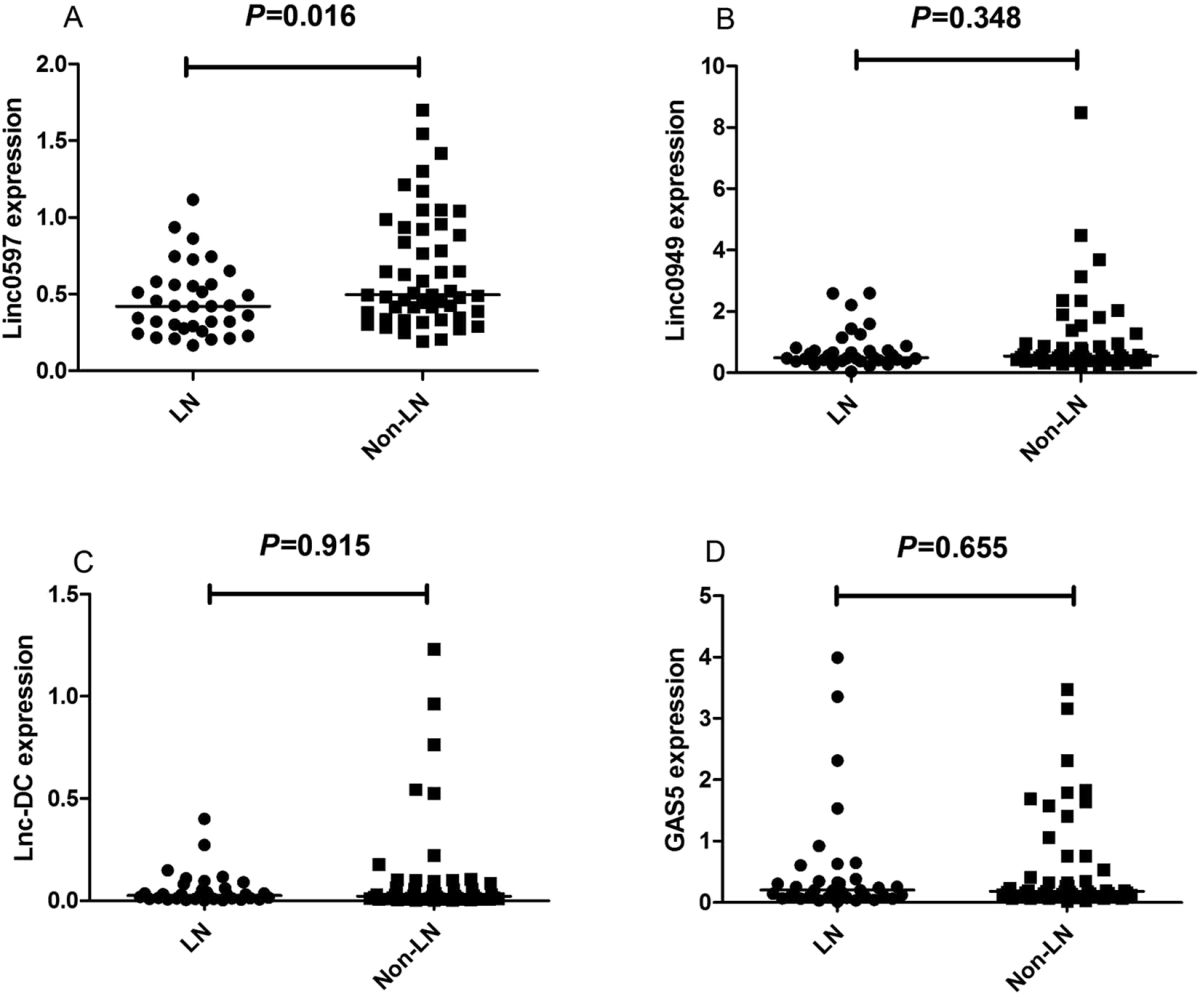
Comparison of expression of lncRNAs between lupus nephritis (LN) group and SLE non-LN group. (**A**) The expression levels of lin0597 in LN compared with non-LN. (**B**) The expression levels of lin0949 in LN compared with non-LN. (**C**) The expression levels of lnc-DC in LN compared with non-LN. (**D**) The expression levels of GAS5 in LN compared with non-LN.

The four lncRNAs (GAS5, lnc-DC, linc0597 and linc0949) expression levels in PBMCs from 85 patients with SLE and 71 healthy controls were shown in Table 1, Fig. 1. Patients with SLE had lower levels of linc0597, lnc-DC and GAS5 than healthy controls (*Z* = −5.984, *P* < 0.001; *Z* = −3.703, *P* < 0.001; *Z* = −2.995, *P* = 0.003 respectively). No significant differences in linc0949 level was found between SLE patients and healthy controls (*Z* = −0.254, *P* = 0.799). When we divided the SLE patients into lupus nephritis (LN) and without nephritis, the expression levels of the four lncRNAs did not show significant difference in LN compared with those without nephritis (all *P* > 0.0125) (Fig. 2).

**Table 1:** Comparison of the lncRNAs expression level between different groups.

Group	Number	Linc0597	Linc0949	Lnc-DC	GAS5
Healthy controls	71	0.81(0.65,1.14)	0.53(0.40,0.88)	0.04(0.03,0.15)	0.27(0.19,0.54)
SLE	85	0.48(0.32,0.74)^a^	0.55(0.42,0.91)	0.02(0.01,0.08)^a^	0.19(0.10,0.47)^b^
LN	35	0.42(0.28,0.56)	0.49(0.38,0.81)	0.03(0.01,0.08)	0.20(0.08,0.38)
Non-LN	50	0.50(0.37,0.93)	0.55(0.42,1.04)	0.02(0.01,0.09)	0.18(0.11,0.59)

All the expression levels were displayed as median value (interquartile range)

^a^ *vs* Healthy controls, *P* < 0.001; ^b^ *vs* Healthy controls, *P* = 0.003; SLE: systemic lupus erythematosus; LN: lupus nephritis.

The associations between lncRNAs levels and clinical features or laboratory parameters of SLE patients were also analyzed. As shown in Tables S5–S6, the expression levels of linc0597 were significantly decreased in patients with proteinuria (*Z* = −2.865, *P* = 0.004). However, no significant association between GAS5 or lnc-DC expression levels with any clinical manifestations or laboratory parameters were identified.

Correlation analysis demonstrated that C-reactive protein (CRP) may be correlated with the expression levels of lnc-DC and GAS5 (all *P* < 0.0125). In addition, disease duration was correlated with the expression level of lnc-DC (*P* = 0.011), but disease activity (SLEDAI-2K), complements 3 (C3) and complements 4 (C4) did not show any correlations with the levels of these lncRNAs (all *P* > 0.0125) (Table 2).

**Table 2:** Correlation between the lncRNAs expression and several clinical parameters of SLE patients.

Parameters	Number	Linc0597		Linc0949		Lnc-DC		GAS5	
		*rs*	*P*	*rs*	*P*	*rs*	*P*	*rs*	*P*
C3	81	0.030	0.793	−0.023	0.836	−0.064	0.573	−0.030	0.794
C4	69	−0.007	0.953	−0.109	0.371	−0.147	0.228	−0.104	0.394
CRP	74	−0.100	0.397	−0.278	0.016	−0.347	0.002	−0.351	0.002
SLEDAI-2K	85	−0.267	0.013	−0.207	0.057	−0.176	0.108	−0.111	0.312
Disease duration	84	−0.109	0.325	−0.159	0.149	−0.277	0.011	−0.234	0.032

SLEDAI-2K: Systemic Lupus Erythematosus Disease Activity Index 2000.

CRP: C-reactive protein; C3 complements 3; C4: complements 4.

Furthermore, the potential influence of the main medical therapies on lncRNAs expression levels were evaluated. However, the expression of these lncRNAs exhibited no significant differences in patients receiving medium to high doses of prednisone (>30 mg/day) compared with patients treated with low doses of prednisone, nor did in the SLE being treated with immunosuppressants (azathioprine, cyclophosphamide, cyclosporine, tacrolimus, leflunomide, mycophenolate mofetil and methotrexate) compared with those without at the time of blood collection (Table 3).

**Table 3:** Influence of medical therapy on lncRNAs expression level in SLE.

Group	Number	Linc0597	Linc0949	Lnc-DC	GAS5
Prednisone (mg/day)					
≥30	28	0.42(0.29,0.90)	0.63(0.42,1.18)	0.03(0.01,0.10)	0.28(0.09,0.64)
<30	56	0.49(0.33,0.65)	0.52(0.41,0.85)	0.02(0.01,0.06)	0.17(0.10,0.33)
Immunosuppressants					
Yes	30	0.47(0.32,0.90)	0.57(0.40,1.64)	0.03(0.01,0.11)	0.22(0.11,0.75)
No	54	0.48(0.33,0.73)	0.52(0.42,0.86)	0.02(0.01,0.06)	0.17(0.08,0.35)

All the expression levels were described as median value (interquartile range).

### Polymorphisms in lncRNAs with SLE risk

Base on the results of lncRNAs expression levels in the first stage, five SNPs (rs10515177 for lnc-DC; rs2070107, rs2632516, rs2877877 for linc0597, rs2067079 for GAS5) were included in association study of polymorphisms in lncRNAs with SLE risk.

First of all, 860 SLE patients and 831 healthy controls were included in phase I, the results of allelic and genotypic frequency for the four SNPs in patients with SLE and health controls were shown in Table S7. A significant association was observed between susceptibility to SLE and the distribution of genotype (CC *vs* GG) at SNP rs2070107 (*P* = 0.007), furthermore, an increased risk was also found in the recessive model (CC *vs* CG + GG) (*P* = 0.007). However, the associations were disappeared after adjustment for gender and age (*P* = 0.025; *P* = 0.022 respectively). In addition, we did not find significant correlations of rs10515177, rs2632516 and rs2877877 genetic polymorphisms with susceptibility to SLE (all *P* > 0.01).

Due to the inconsistent results after adjustment for gender and age in phase I, another independent set of 400 SLE cases and 400 healthy controls were recruited to verify our previous results. The results of allelic and genotypic frequency for the four SNPs in patients with SLE and health controls were shown in Table S8. The final results showed that the distribution of genotype (CG *vs* GG), allele (C *vs* G) and dominant model (CC + CG *vs* GG) of rs2070107 was associated with SLE (all *P* < 0.01). The distribution of genotype (GG *vs* AA, GA *vs* AA), allele (G *vs* A) and dominant model (GG + GA *vs* AA) of rs2877877 was associated with SLE (all *P* < 0.01). But rs10515177, rs2632516 did not show significant association with SLE (all *P* > 0.01). Since the *P*-value of Hardy-Weinberg equilibrium (HWE) in health controls was <0.01, we did not take rs2067079 into consideration.

At last, we combined the results of the two phase, however, our meta-analysis results (1260 SLE patients and 1231 healthy controls) indicated that there was no obvious relationship between the polymorphisms of the five SNPs (rs10511577, rs2067079, rs2070107, rs2632516, rs2877877) and susceptibility to SLE (all *P* > 0.01), Table 4.

**Table 4:** Allele and genotype frequencies of five SNPs in SLE patients and health controls.

SNPs	Group	Phase I^a^		Phase II^a^		Meta-analysis			heterogeneity	
		*OR(95% CI)*	*P*	*OR(95% CI)*	*P*	*OR(95% CI)*	*P*	*Model*	*I* ^*2*^ *(%)*	*P*
rs10515177	Genotype									
	GG vs. AA	0.818(0.279–2.399)	0.714	1.478(0.584–3.744)	0.410	1.169(0.590–2.323)	0.654	F	0.0	0.398
	GA vs. AA	0.772(0.584–1.021)	0.069	0.928(0.628–1.370)	0.706	0.836(0.670–1.043)	0.113	F	0.0	0.393
	Allele									
	G vs. A	0.802(0.626–1.027)	0.080	1.069(0.776–1.474)	0.683	0.893(0.734–1.086)	0.256	F	48.5	0.163
	Dominant model									
	GG + GA vs. AA	0.774(0.590–1.017)	0.066	0.987(0.684–1.424)	0.945	0.859(0.694–1.063)	0.163	F	24.4	0.250
	Recessive model									
	GG vs. GA + AA	0.852(0.291–2.496)	0.770	1.496(0.592–3.781)	0.394	1.193(0.604–2.360)	0.611	F	0.0	0.422
rs2070107	Genotype									
	CC vs. GG	2.111(1.097–4.062)	0.025	0.646(0.277–1.506)	0.312	1.314(0.376–4.588)	0.669	R	82.1	0.018
	CG vs. GG	0.959(0.765–1.203)	0.719	0.620(0.448–0.858)	0.004	0.817(0.536–1.245)	0.347	R	79.2	0.028
	Allele									
	C vs. G	1.156(0.960–1.393)	0.127	0.702(0.538–0.916)	0.009	0.909(0.558–1.482)	0.703	R	89.0	0.003
	Dominant model									
	CC + CG vs. GG	1.031(0.828–1.283)	0.787	0.623(0.456–0.852)	0.003	0.849(0.516–1.396)	0.519	R	86.0	0.007
	Recessive model									
	CC vs. CG + GG	2.137(1.114–4.098)	0.022	0.741(0.319–1.719)	0.485	1.402(0.454–4.333)	0.557	R	78.2	0.032
rs2632516	Genotype									
	GG vs. CC	0.812(0.614–1.072)	0.141	1.189(0.786–1.797)	0.413	0.968(0.650–1.442)	0.872	R	63.2	0.099
	GC vs. CC	0.835(0.652–1.070)	0.155	1.132(0.781–1.641)	0.513	0.927(0.758–1.134)	0.460	F	46.1	0.173
	Allele									
	G vs. C	0.904(0.789–1.035)	0.143	1.101(0.902–1.344)	0.345	0.983(0.812–1.191)	0.864	R	61.2	0.108
	Dominant model									
	GG + GC vs. CC	0.827(0.654–1.045)	0.111	1.152(0.811–1.637)	0.430	0.963(0.692–1.341)	0.823	R	61.9	0.105
	Recessive model									
	GG vs. GC + CC	0.919(0.738–1.144)	0.449	1.089(0.791–1.500)	0.601	0.971(0.814–1.159)	0.747	F	5.3	0.304
rs2877877	Genotype									
	GG vs. AA	1.197(0.817–1.754)	0.355	0.502(0.301–0.836)	0.008	0.822(0.337–2.004)	0.666	R	87.8	0.004
	GA vs. AA	0.905(0.737–1.112)	0.343	0.615(0.452–0.836)	0.002	0.787(0.529–1.172)	0.239	R	79.5	0.027
	Allele									
	G vs. A	1.044(0.898–1.213)	0.579	0.675(0.543–0.840)	<0.001	0.846(0.552–1.296)	0.441	R	90.3	0.001
	Dominant model									
	GG + GA vs. AA	0.946(0.777–1.152)	0.580	0.591(0.442–0.790)	<0.001	0.786(0.485–1.274)	0.328	R	87.4	0.005
	Recessive model									
	GG vs. GA + AA	1.252(0.864–1.813)	0.234	0.624(0.382–1.020)	0.060	0.923(0.453–1.881)	0.826	R	82.2	0.018
rs2067079	Genotype									
	TT vs. CC	0.906(0.662–1.241)	0.538	0.397(0.235–0.672)	0.001	0.611(0.282–1.323)	0.212	R	84.9	0.010
	TC vs. CC	1.142(0.927–1.408)	0.213	0.412(0.300–0.565)	<0.001	0.720(0.269–1.933)	0.515	R	96.5	<0.001
	Allele									
	T vs. C	1.005(0.872–1.159)	0.945	0.618(0.503–0.760)	<0.001	0.793(0.493–1.276)	0.339	R	93.1	<0.001
	Dominant model									
	TT + TC vs. CC	1.085(0.891–1.322)	0.416	0.409(0.301–0.557)	<0.001	0.693(0.273–1.764)	0.442	R	96.3	<0.001
	Recessive model									
	TT vs. TC + CC	0.847(0.630–1.140)	0.274	0.690(0.425–1.119)	0.133	0.770(0.601–0.987)	0.039	F	0.0	0.512

^a^Results of Genotype, Dominant and Recessive model were adjusted by gender and age; SLE: systemic lupus erythematosus; HC: health controls; R:random-effects model; F:fixed-effects model.

### Association of lncRNAs expression levels with their genotypes in patients with SLE

65 SLE patients were recruited to examine the associations between lncRNAs expression levels and their respective genotypes (Table 5). However, no significant differences in expression levels of lncRNAs were observed between SLE with different genotypes (all *P* > 0.01).

**Table 5:** Association of lncRNAs expression levels with genotypes at 5 SNPs.

SNPs	Genotype	Number	LncRNAs expression leve*l*	*P*
rs10515177^a^	GG	1	1.83	0.386
	GA	11	0.57(0.16–1.04)	
	AA	51	0.29(0.07–0.51)	
rs2070107^b^	CC	1	0.69	0.945
	CG	15	0.01(0.00–1.87)	
	GG	47	0.01(0.00–1.90)	
rs2632516^b^	GG	20	0.00(0.00–0.01)^d^	0.151
	GC	31	1.15(0.01–2.31)	
	CC	13	0.01(0.00–1.71)	
rs2877877^b^	GG	7	0.01(0.00–0.69)	0.305
	GA	24	1.26(0.00–2.12)	
	AA	33	0.01(0.00–1.75)	
rs2067079^c^	TT	6	0.29(0.23–0.39)	0.068
	TC	27	0.66(0.37–0.90)	
	CC	32	0.54(0.31–1.27)	

All the expression levels were displayed as median value (interquartile range).

^a^Lnc-DC expression level; ^b^Linc0597 expression level; ^c^GAS5 expression level;

^d^0.004(0.003–0.008) in three decimal place.

## Discussion

Nowadays, increasing evidence has shown that lncRNAs may play a major biological role in physiological processes that maintain cellular and tissue homeostasis^26^. And, lncRNAs, which have been primarily studied in the context of genomic imprinting and cell differentiation, are now emerging as key regulators of diverse biological process especially by immune cells and the molecular mechanism of autoimmunity. Recent studies have suggested that lncRNAs might be associated with numerous autoimmune diseases^10,14^, suggesting that lncRNAs may open a new avenue for SLE study.

In the current study, according to the available evidence and our recent study on the plasma expression of lncRNAs^25^, we detected the expression levels of four lncRNAs (GAS5, lnc-DC, linc0597 and linc0949) in the PBMCs from SLE patients at the first stage, and investigated their clinical associations. Our results demonstrated that the expression of linc0597, lnc-DC and GAS5 were decreased in patients with SLE than healthy controls, however, linc0949, which was reported at low level in PBMCs of patients with SLE in a recent study^20^, showed no significant differences in our study. One explanation is that linc0949 expression was influenced by medical treatment. The other reason may be the different internal control used between their study and our study. At last, the results of expression levels of four lncRNAs in the PBMCs from SLE patients were roughly consistent with our previous study in the plasma^25^.

Then, polymorphisms in lncRNAs (rs10515177 for lnc-DC; rs2070107, rs2632516, rs2877877 for linc0597, rs2067079 for GAS5) with SLE risk were analyzed. Our pooled results revealed that there was no obvious relationship between the polymorphisms of the five SNPs and susceptibility to SLE. Besides, we have tried to detect the associations of lncRNAs expression levels with their respective genotypes in SLE patients, but no significant differences were observed.

The existing evidences suggest that activation, differentiation, and imbalance expression of immune cells, such as T cells, B cells, macrophages, and NK cells alter the autoimmunity which may have direct link to lncRNAs^27^. There were also evidence that lncRNAs can be regulated through the stimulators of toll-like receptors (TLRs)^28–30^, and TLRs have an important role in the pathogenesis of SLE^31,32^. In addition, tumor necrosis factor-α (TNF-α) play crucial roles in defense against inflammatory and immune responses of SLE^33,34^. LncRNAs and their binding proteins can regulate TNF-α expression and thus may play important roles in the innate immune response and inflammatory diseases in humans^28^. Last but not least, Zhang *et al*.^19^ indicated that lncRNA NEAT1 could affect the late mitogen-activated protein kinase (MAPK) pathway activation and consequently regulate a set of lipopolysaccharide (LPS)-induced cytokines and chemokines which were dysregulated in patients with SLE, and this demonstrated that lncRNAs may contribute to a new layer of molecular regulation of autoimmune diseases.

Several limitations should be acknowledged in this study. First of all, selection bias may be existed, especially in the choosing of healthy controls. Second, only conservative Bonferroni correction and Logistic regression were chosen during the comparison in multiple groups or mismatch factors between the case and control. Finally, we only have 80% power to detect genetic effects at an OR > 1.85 or OR <0.80 in our current total samples, therefore, part of our analysis may be under-powered.

In summary, the expression levels of linc0597, lnc-DC and GAS5 were down-regulated in SLE patients, but their gene polymorphisms with SLE and the associations between lncRNAs expression levels with the respective genotypes in SLE patients still need further studies.

## Materials and Methods

### Patients and healthy controls

A two stage case-control studies were conducted in a Han Chinese population. Briefly, 85 SLE patients and 71 healthy controls were enrolled to investigate the expression levels of GAS5 (ENST00000449289), lnc-DC (ENST00000587298), linc0597 (ENST00000500597) and linc0949 (ENST00000500949) in PBMCs in stages one. Then, 1260 SLE patients (phase I: 860 SLE patients; phase II: 400 SLE patients) and 1231 healthy controls (phase I: 831 healthy controls; phase II: 400 healthy controls) were included to detect the single nucleotide polymorphisms (SNPs) in the differentially expressed lncRNAs in stage two. All of these SLE patients were recruited from Anhui Provincial Hospital and the First Affiliated Hospital of Anhui Medical University, and the healthy controls were recruited from the physical examination center of the Second Affiliated Hospital of Anhui Medical University and health blood donors. All the patients with SLE were diagnosed according to the American College of Rheumatology (ACR) diagnostic criteria revised in 1997^35^. The severity of disease was assessed with the systemic lupus erythematosus disease activity index 2000 (SLEDAI-2K)^36^. The study was approved by the Medical Ethics Committee of Anhui Medical University. Methods were carried out in accordance with the approved guidelines. All subjects were enrolled after informed consent had been obtained.

### Extraction of RNA and quantitative real-time reverse transcription polymerase chain reaction (qRT-PCR)

Peripheral blood samples (5 ml) were collected in tubes containing ethylenediaminetetraacetic acid (EDTA) from each subject. PBMCs were purified from peripheral blood by Ficoll-Hypaque density gradient centrifugation. Total RNA was extracted from PBMCs using TRIzol reagent (Invitrogen, Carlsbad, CA, USA) and the concentrations of RNA were measured by a NanoDrop™ 2000 spectrophotometer (Thermo Scientific, USA).

Total RNA were reverse-transcribed into cDNA by the PrimeScript^TM^ RT reagent Kit (Takara Bio Inc, Japan). To determine the expression level, quantitative real-time PCR (qPCR) with SYBR Green (SYBR® Premix Ex Taq™ II,Takara Bio Inc, Japan) was performed using an ABI ViiA™ 7 Real-Time PCR System (Applied Biosystems, Foster City, CA, USA). Cycle conditions were as follows: 95 °C for 1 min, followed by 42 cycles at 95 °C for 10 sec, 60 °C for 30 sec and 72 °C for 1 min. The lncRNA expression was determined by comparison with housekeeping gene β-actin from the same sample as internal control. The primer sequences used for qPCR are given in Table S9. The relative expression of lncRNAs were calculated using 2^−△△Ct^ method normalized to endogenous control^37^.

### SNP selection and genotyping

The genetic and location information were verified through the database of LNCipedia.org (v4.0) and Genome Browser Gateway (UCSC). Using genotype data of Han Chinese in Beijing from HapMap database (HapMap Data Rel 24/Phase II, Nov 08, on NCBI B36 assembly, dbSNP b126) and Ensembl genome browser 85, we selected five tagSNPs (1 for GAS5, 3 for linc0597,1 for lnc-DC) capturing all the common SNPs (minor allele frequency, MAF > 0.05) located in the chromosome locus transcribed into those lncRNA and their flanking 2000 bp region. The selection was conducted with the pairwise option of the Haploview 4.0 software (Cambridge, MA, USA) and the threshold for analyses was set as *r* ^2^ > 0.8. Overall flow of SNP selection of the five selected tagSNPs were summarized in Figures S1–S3.

The genomic DNA was prepared from the peripheral blood leukocytes according to the standard procedures with the Flexi Gene-DNA Kit (Qiagen, Valencia, CA).The genotyping was conducted using TaqMan SNP genotyping assays by an EP1 platform (Fluidigm, South San Francisco, CA, USA). Only those individuals with 100% genotype success for all markers were included for final analysis.

### Statistical analysis

Normally distributed data were expressed as mean ± SD, nonnormality distribution data were expressed as median value and interquartile range (IQR). Categorical variables were represented by frequency and percentage. The nonparametric test was used to compare gene expression between groups, and the correlation between groups was evaluated by Spearman’s rank correlation coefficient test. For the allelic association of each polymorphism with SLE susceptibility was assessed with chi-square (*χ* ^2^) test. The genotype frequencies of the SNPs were tested for HWE in control subjects. Logistic regression analysis was chosen to adjust the gender and age which were not matched well between the SLE and health controls, variables were entered into the multivariate model. Two models were used for statistical analysis, including dominant model (homozygous rare + heterozygous *vs* homozygous frequent allele), recessive model (homozygous rare *vs* heterozygous + homozygous frequent allele)^38,39^.

General statistical analysis was performed by the SPSS 20.0 software (IBM Corp., Armonk, NY, USA), meta-analysis was conducted by the Stata 12.0 software (Stata Corporation, College Station, TX, USA). Figures were generated by GraphPad Prism version 5.0 (GraphPad Software, La Jolla, CA, USA). Bonferroni correction was considered in this study, we used a significance threshold of 0.0125 (0.05/4) in the analysis of expression levels about the four lncRNAs in stages one, and a significance threshold of 0.010 (0.05/5) was applied in the detection of SNPs in stage two.

## Electronic supplementary material

Supplementary materials
