# Supplementary material for: Association of long noncoding RNAs expression levels and their gene polymorphisms with systemic lupus erythematosus
Source: Sci Rep. 2017 Nov 9;7:15119. doi: 10.1038/s41598-017-15156-4 (PMC5680319; doi:10.1038/s41598-017-15156-4)
Supplement: Supplementary file 1 — Supplementary materials [file 41598_2017_15156_MOESM1_ESM.pdf]

Association of long noncoding RNAs expression levels and their gene polymorphisms with systemic lupus erythematosus

Jun Li[1,2,3#], Guo-Cui Wu[1,2#], Tian-Ping Zhang[1,2], Xiao-Ke Yang[1,2], Shuang-Shuang Chen[1,2], Lian-Ju Li[1,2], Shu-Zhen Xu[1,2], Tian-Tian Lv[1,2], Rui-Xue Leng[1,2], Hai-Feng Pan[1,2*], Dong-Qing Ye[1,2*]

[1] Department of Epidemiology and Biostatistics, School of Public Health, Anhui Medical University, Anhui, P.R.China.

[2] Anhui Province Key Laboratory of Major Autoimmune Diseases, Anhui, P.R.China.

[3] Jiangyin Center for Disease Control and Prevention, Jiangsu, P.R.China.

[#]Jun Li and Guo-Cui Wu contributed equally to this work and should be considered co-first authors.

[*]**Correspondence:**

Dong-Qing Ye (ydqahmu@gmail.com, ydq@ahmu.edu.cn);

Hai-Feng Pan (panhaifeng@ahmu.edu.cn, panhaifeng1982@sina.com)

Address: Department of Epidemiology and Biostatistics, School of Public Health, Anhui Medical University, 81 Meishan Road, Hefei, Anhui, 230032, PR China, Tel: +86 551 65167726; Fax: +86 551 65161171

Table S1 Characteristics of the SLE patients and healthy controls in stage one

Characteristics	SLE patients	Healthy controls
Demographic characteristics	85	71
Age (years),M(P_{25},P_{75})	38(26,47)	40(26,51)
Sex (female/male)	77/8	62/9
Disease duration (years),M(P_{25},P_{75})	4.74(0.69,9.06)	-
SLEDAI-2K,M(P_{25},P_{75})	12(7,19)	-
Clinical manifestations		
Lupus nephritis, n (%)	35(41.2)	-
Arthritis, n (%)	24(28.2)	-
Myositis, n (%)	7(8.2)	-
Rash, n (%)	40(47.1)	-
Alopecia, n (%)	29(34.1)	-
Oral ulcer, n (%)	16(18.8)	-
Pleuritis, n (%)	8(9.4)	-
Fever, n (%)	29(34.1)	-
Vision disorder , n (%)	8(9.4)	-
Laboratory measurements		
Anti-dsDNA, n (%)	36(42.4)	-
Anti-Sm, n (%)	33(39.8)	-
Anti-SSA, n (%)	61(74.4)	-
Anti-SSB, n (%)	7(8.5)	-
Anti-RNP, n (%)	31(37.8)	-
Anti-Ribosomal P, n (%)	25(30.5)	-
Leukopenia, n (%)	14(16.5)	-
Thrombocytopenia, n (%)	29(34.1)	-
Hematuria, n (%)	36(42.4)	-
Proteinuria, n (%)	42(49.4)	-
Low complement, n (%)	56(65.9)	-
Medical therapy		
Prednisone(≥30mg/day)	28(33.3)	-
Immunosuppressants	30(35.7)	-

SLEDAI-2K: Systemic Lupus Erythematosus Disease Activity Index 2000; dsDNA: double-stranded DNA; Sm: Smith; SSA: Sjögren's syndrome-related antigen A; SSB: Sjögren's syndrome-related antigen B; RNP: Ribonucleoprotein; Immunosuppressants: azathioprine, cyclophosphamide, cyclosporine,tacrolimus, leflunomide, mycophenolate mofetil and methotrexate

Table S2 Characteristics of the SLE patients and healthy controls in stage two (phase I)

SNP	Number		Characteristics	SLE patients	Healthy controls	t/χ^2	P	P_{HWE} (in HC)
	SLE	HC						
rs10515177	856	736	Age	37.39±12.76	38.06±16.68	0.887	0.375	0.916
			Sex (female/male)	790/66	566/170	74.210	<0.001	
rs2067079	850	831	Age	37.49±12.77	38.61±16.39	1.559	0.119	0.017
			Sex (female/male)	786/64	660/171	59.491	<0.001	
rs2070107	857	729	Age	37.44±12.79	38.30±16.77	1.128	0.260	0.121
			Sex (female/male)	790/67	564/165	69.239	<0.001	
rs2632516	860	825	Age	37.47±12.79	38.69±16.45	1.684	0.092	0.508
			Sex (female/male)	792/68	656/169	54.834	<0.001	
rs2877877	850	819	Age	37.48±12.76	38.28±16.31	1.109	0.268	0.128
			Sex (female/male)	783/67	643/176	61.781	<0.001	

HC:health control; HWE: Hardy-Weinberg equilibrium

Table S3 Characteristics of the SLE patients and healthy controls in stage two (phase II)

SNP	Number		Characteristics	SLE patients	Healthy controls	t/χ^2	P	P_{HWE} (in HC)
	SLE	HC						
rs10515177	396	396	Age	36.74±11.00	39.99±16.55	3.255	0.001	0.035
			Sex (female/male)	373/23	396/0	23.688	<0.001	
rs2067079	392	397	Age	36.77±10.95	39.90±16.52	3.138	0.002	<0.001
			Sex (female/male)	370/22	397/0	22.920	<0.001	
rs2070107	394	394	Age	36.78±11.00	39.90±16.58	3.109	0.002	0.551
			Sex (female/male)	371/23	394/0	23.692	<0.001	
rs2632516	392	386	Age	36.81±10.93	40.15±16.51	3.312	0.001	0.809
			Sex (female/male)	369/23	386/0	23.338	<0.001	
rs2877877	391	383	Age	36.84±10.98	40.06±16.63	3.174	0.002	0.847
			Sex (female/male)	369/22	383/0	22.180	<0.001	

HC:health control; HWE: Hardy-Weinberg equilibrium

Table S4 Characteristics of the SLE patients and healthy controls in stage two (pooled)

SNP	Number		Characteristics	SLE patients	Healthy controls	t/χ^2	P	P_{HWE} (in HC)
	SLE	HC						
rs10515177	1252	1132	Age	37.19±12.23	38.74±16.65	2.567	0.010	0.167
			Sex (female/male)	1163/89	962/170	38.401	<0.001	
rs2067079	1242	1228	Age	37.27±12.23	39.03±16.44	3.022	0.003	0.191
			Sex (female/male)	1156/86	1057/171	32.463	<0.001	
rs2070107	1251	1123	Age	37.23±12.26	38.86±16.71	2.676	0.008	0.133
			Sex (female/male)	1161/90	958/165	34.706	<0.001	
rs2632516	1252	1211	Age	37.37±12.24	39.15±16.48	3.212	0.001	0.497
			Sex (female/male)	1161/91	1042/169	29.009	<0.001	
rs2877877	1241	1202	Age	37.28±12.22	38.85±16.43	2.668	0.008	0.348
			Sex (female/male)	1152/89	1026/176	35.076	<0.001	

HC:health control; HWE: Hardy-Weinberg equilibrium

Table S5 Associations between lncRNAs expression level with clinical features of SLE patients

Characteristics	Number	Linc0597	Linc0949	Lnc-DC	GAS5
Arthritis					
+	24	0.46(0.39,0.78)	0.56(0.42,0.85)	0.03(0.01,0.09)	0.17(0.10,0.57)
-	61	0.48(0.31,0.69)	0.51(0.42,0.95)	0.02(0.01,0.08)	0.19(0.10,0.47)
Myositis					
+	7	0.74(0.49,0.94)	0.55(0.49,1.44)	0.03(0.01,0.40)	0.27(0.08,0.76)
-	78	0.46(0.32,0.67)	0.53(0.42,0.89)	0.02(0.01,0.08)	0.19(0.10,0.44)
Rash					
+	40	0.44(0.31,0.74)	0.50(0.39,0.80)	0.02(0.01,0.06)	0.14(0.09,0.33)
-	45	0.51(0.34,0.87)	0.57(0.44,1.10)	0.03(0.01,0.10)	0.20(0.14,0.62)
Alopecia					
+	29	0.42(0.31,0.73)	0.57(0.41,1.01)	0.03(0.01,0.10)	0.26(0.11,0.70)
-	56	0.49(0.33,0.83)	0.52(0.42,0.93)	0.02(0.01,0.06)	0.17(0.09,0.37)
Oral ulcer					
+	16	0.38(0.31,0.70)	0.43(0.34,0.68)	0.01(0.01,0.08)	0.14(0.07,0.53)
-	69	0.49(0.33,0.76)	0.56(0.43,0.95)	0.03(0.01,0.08)	0.20(0.11,0.47)
Pleuritis					
+	8	0.61(0.29,0.85)	0.64(0.46,1.58)	0.05(0.01,0.09)	0.27(0.12,1.77)
-	77	0.46(0.33,0.74)	0.52(0.41,0.87)	0.02(0.01,0.08)	0.18(0.10,0.40)
Vision disorder					
+	8	0.40(0.29,0.48)	0.46(0.41,0.58)	0.02(0.01,0.03)	0.21(0.11,0.26)
-	77	0.49(0.32,0.78)	0.55(0.42,1.05)	0.03(0.01,0.09)	0.19(0.10,0.62)
Fever					
+	29	0.48(0.30,0.79)	0.52(0.39.1.36)	0.02(0.01,0.11)	0.14(0.09,0.76)
-	56	0.47(0.32,0.74)	0.55(0.42,0.87)	0.03(0.01,0.08)	0.20(0.10,0.37)

All the expression levels were displayed as median value (interquartile range)

Table S6 Associations between lncRNAs expression level with laboratory parameters of SLE

Group	Number	Linc0597	Linc0949	Lnc-DC	GAS5
Anti-dsDNA					
+	36	0.42(0.29,0.78)	0.49(0.41,0.80)	0.02(0.01,0.05)	0.18(0.10,0.54)
-	49	0.51(0.34,0.73)	0.57(0.42,0.95)	0.02(0.01,0.09)	0.20(0.11,0.47)
Anti-Sm					
+	33	0.46(0.29,0.87)	0.55(0.42,0.84)	0.02(0.01,0.06)	0.16(0.08,0.37)
-	50	0.49(0.33,0.67)	0.51(0.40,1.18)	0.02(0.01,0.09)	0.20(0.10,0.66)
Anti-SSA					
+	61	0.49(0.34,0.76)	0.57(0.41,0.95)	0.02(0.10,0.09)	0.20(0.10,0.57)
-	21	0.38(0.27,0.65)	0.47(0.42,1.21)	0.02(0.01,0.06)	0.16(0.10,0.55)
Anti-SSB					
+	7	0.75(0.58,1.30)	0.95(0.62,2.59)[a]	0.10(0.02,0.54)	0.53(0.11,3.48)
-	75	0.45(0.32,0.65)	0.50(0.41,0.81)	0.02(0.01,0.06)	0.18(0.10,0.35)
Anti-RNP					
+	31	0.48(0.33,0.84)	0.50(0.42,0.95)	0.02(0.01,0.06)	0.18(0.08,0.61)
-	51	0.46(0.30,0.65)	0.56(0.41,0.95)	0.02(0.01,0.09)	0.20(0.10,0.53)
Anti-Ribosomal P					
+	25	0.45(0.35,0.76)	0.50(0.40,1.05)	0.02(0.01,0.07)	0.20(0.08,0.70)
-	57	0.48(0.32,0.70)	0.52(0.42,0.84)	0.02(0.01,0.09)	0.18(0.10,0.36)
Leukopenia					
+	14	0.60(0.45,0.91)	0.55(0.41,1.17)	0.03(0.01,0.10)	0.18(0.08,0.85)
-	71	0.45(0.32,0.65)	0.52(0.42,0.87)	0.02(0.01,0.08)	0.19(0.10,0.41)
Thrombocytopenia					
+	29	0.45(0.31,0.94)	0.50(0.41,1.05)	0.02(0.01,0.09)	0.20(0.11,0.63)
-	56	0.48(0.34,0.65)	0.56(0.42,0.85)	0.02(0.01,0.08)	0.18(0.09,0.37)
Hematuria					
+	36	0.46(0.28,0.65)	0.52(0.38,0.85)	0.02(0.01,0.09)	0.18(0.08,0.62)
-	49	0.48(0.35,0.86)	0.55(0.42,0.95)	0.02(0.01,0.07)	0.20(0.10,0.40)
Proteinuria					
+	42	0.40(0.29,0.57)[a]	0.50(0.42,0.74)	0.02(0.01,0.06)	0.18(0.09,0.36)
-	43	0.52(0.42,0.96)	0.56(0.42,1.39)	0.02(0.01,0.10)	0.19(0.10,0.76)
Low complement					
+	56	0.44(0.32,0.74)	0.52(0.41,0.95)	0.02(0.01,0.08)	0.18(0.10,0.59)
-	29	0.52(0.33,0.83)	0.56(0.43,0.87)	0.03(0.01,0.09)	0.19(0.11,0.36)
Elevated ESR(>20g/L)					
+	51	0.48(0.29,0.65)	0.50(0.42,0.81)	0.02(0.01,0.06)	0.16(0.08,0.33)
-	29	0.45(0.34,0.69)	0.56(0.41,1.22)	0.02(0.01,0.09)	0.20(0.13,0.84)

All the expression levels were displayed as median value (interquartile range)

dsDNA:double-stranded DNA; Sm: Smith; SSA: Sjögren's syndrome-related antigen A; SSB: Sjögren's syndrome-related antigen B; RNP: Ribonucleoprotein; ESR: erythrocyte sedimentation rate

[a] Laboratory parameter (+) *vs* Laboratory parameter (-), $P<0.0125$.

Table S7 Allele and genotype frequencies of five SNPs in SLE patients and health controls in stage two (phase I)

SNPs	Group	SLE	HC	Crude			Adjusted		
				χ^2	*OR*(95% *CI*)	*P*	χ^2	*OR*(95% *CI*)	*P*
rs10515177	Genotype								
	GG	7(0.8)	7(1.0)	0.127	0.826(0.288-2.367)	0.722	0.134	0.818(0.279-2.399)	0.714
	GA	120(14.0)	127(17.2)	3.200	0.780(0.595-1.024)	0.074	3.300	0.772(0.584-1.021)	0.069
	AA	729(85.2)	602(81.8)		1.000			1.000	
	Allele								
	G	134(7.8)	141(9.6)	3.068	0.802(0.626-1.027)	0.080			
	A	1578(92.2)	1331(90.4)		1.000				
	Dominant model								
	GG+GA	127(14.8)	134(18.2)	3.269	0.783(0.600-1.021)	0.071	3.375	0.774(0.590-1.017)	0.066
	AA	729(85.2)	602(81.8)		1.000			1.000	
	Recessive model								
	GG	7(0.8)	7(1.0)	0.081	0.859(0.300-2.459)	0.777	0.085	0.852(0.291-2.496)	0.770
	GA+AA	849(99.2)	729(99.0)		1.000			1.000	
rs2070107	Genotype								
	CC	36(4.2)	13(1.8)	7.164	2.414(1.266-4.601)	0.007	5.000	2.111(1.097-4.062)	0.025
	CG	237(27.7)	207(28.4)	0.000	0.998(0.800-1.245)	0.985	0.129	0.959(0.765-1.203)	0.719
	GG	584(68.1)	509(69.8)		1.000			1.000	
	Allele								
	C	309(18.0)	233(16.0)	2.328	1.156(0.960-1.393)	0.127			
	G	1405(82.0)	1225(84.0)		1.000				
	Dominant model								
	CC+CG	273(31.9)	220(30.2)	0.517	1.082(0.873-1.339)	0.472	0.073	1.031(0.828-1.283)	0.787
	GG	584(68.1)	509(69.8)		1.000			1.000	

	Recessive model								
	CC	36(4.2)	13(1.8)	7.244	2.415(1.271-4.590)	0.007	5.220	2.137(1.114-4.098)	0.022
	CG+GG	821(95.8)	716(98.2)		1.000			1.000	
rs2632516	Genotype								
	GG	225(26.2)	231(28.0)	2.276	0.811(0.618-1.065)	0.131	2.163	0.812(0.614-1.072)	0.141
	GC	426(49.5)	420(50.9)	1.874	0.844(0.663-1.076)	0.171	2.027	0.835(0.652-1.070)	0.155
	CC	209(24.3)	174(21.1)		1.000			1.000	
	Allele								
	G	876(50.9)	882(53.5)	2.150	0.904(0.789-1.035)	0.143			
	C	844(49.1)	768(46.5)		1.000				
	Dominant model								
	GG+GC	651(75.7)	651(78.9)	2.469	0.833(0.662-1.046)	0.116	2.536	0.827(0.654-1.045)	0.111
	CC	209(24.3)	174(21.1)		1.000			1.000	
	Recessive model								
	GG	225(26.2)	231(28.0)	0.720	0.911(0.735-1.130)	0.396	0.572	0.919(0.738-1.144)	0.449
	GC+CC	635(73.8)	594(72.0)		1.000			1.000	
rs2877877	Genotype								
	GG	73(8.6)	55(6.7)	1.616	1.275(0.877-1.855)	0.204	0.854	1.197(0.817-1.754)	0.355
	GA	343(40.3)	347(42.4)	0.253	0.950(0.777-1.161)	0.615	0.897	0.905(0.737-1.112)	0.343
	AA	434(51.1)	417(50.9)		1.000			1.000	
	Allele								
	G	489(28.8)	457(27.9)	0.307	1.044(0.898-1.213)	0.579			
	A	1211(71.2)	1181(72.1)		1.000				
	Dominant model								
	GG+GA	416(48.9)	402(49.1)	0.003	0.994(0.821-1.205)	0.953	0.307	0.946(0.777-1.152)	0.580
	AA	434(51.1)	417(50.9)		1.000			1.000	

	Recessive model								
	GG	73(8.6)	55(6.7)	2.056	1.305(0.907-1.878)	0.152	1.414	1.252(0.864-1.813)	0.234
	GA+AA	777(91.4)	764(93.3)		1.000			1.000	
rs2067079	Genotype								
	TT	97(11.4)	114(13.7)	0.647	0.882(0.649-1.198)	0.421	0.379	0.906(0.662-1.241)	0.538
	TC	393(46.2)	344(41.4)	2.607	1.184(0.965-1.453)	0.106	1.554	1.142(0.927-1.408)	0.213
	CC	360(42.4)	373(44.9)		1.000			1.000	
	Allele								
	T	587(34.5)	572(34.4)	0.005	1.005(0.872-1.159)	0.945			
	C	1113(65.5)	1090(65.6)		1.000				
	Dominant model								
	TT+TC	490(57.6)	458(55.1)	1.096	1.109(0.914-1.344)	0.295	0.660	1.085(0.891-1.322)	0.416
	CC	360(42.4)	373(44.9)		1.000			1.000	
	Recessive model								
	TT	97(11.4)	114(13.7)	2.032	0.810(0.607-1.082)	0.154	1.198	0.847(0.630-1.140)	0.274
	TC+CC	753(88.6)	717(86.3)		1.000			1.000	

Adjusted by gender and age; SLE: systemic lupus erythematosus; HC: health controls

Table S8 Allele and genotype frequencies of five SNPs in SLE patients and health controls in stage two (phase II)

SNPs	Group	SLE	HC	Crude			Adjusted		
				χ^2	*OR*(95% *CI*)	*P*	χ^2	*OR*(95% *CI*)	*P*
rs10515177	Genotype								
	GG	12(3.0)	8(2.0)	0.788	1.505(0.607-3.730)	0.378	0.680	1.478(0.584-3.744)	0.410
	GA	62(15.7)	65(16.4)	0.052	0.957(0.654-1.400)	0.820	0.142	0.928(0.628-1.370)	0.706
	AA	322(81.3)	323(81.6)		1.000			1.000	
	Allele								
	G	86(10.9)	81(10.2)	0.167	1.069(0.776-1.474)	0.683			
	A	706(89.1)	711(89.8)		1.000				
	Dominant model								
	GG+GA	74(18.7)	73(18.4)	0.008	1.017(0.711-1.455)	0.927	0.005	0.987(0.684-1.424)	0.945
	AA	322(81.3)	323(81.6)		1.000			1.000	
	Recessive model								
	GG	12(3.0)	8(2.0)	0.810	1.516(0.613-3.749)	0.368	0.726	1.496(0.592-3.781)	0.394
	GA+AA	384(97.0)	388(98.0)		1.000			1.000	
rs2070107	Genotype								
	CC	10(2.5)	13(3.3)	0.846	0.674(0.290-1.563)	0.358	1.024	0.646(0.277-1.506)	0.312
	CG	94(23.9)	127(32.2)	7.254	0.648(0.473-0.889)	0.007	8.318	0.620(0.448-0.858)	0.004
	GG	290(37.6)	254(64.5)		1.000			1.000	
	Allele								
	C	114(14.5)	153(19.4)	6.817	0.702(0.538-0.916)	0.009			
	G	674(85.5)	635(80.6)		1.000				
	Dominant model								
	CC+CG	104(26.4)	140(35.5)	7.651	0.651(0.480-0.882)	0.006	8.820	0.623(0.456-0.852)	0.003

	GG	290(73.6)	254(64.5)		1.000			1.000	
	Recessive model								
	CC	10(2.5)	13(3.3)	0.401	0.763(0.331-1.762)	0.527	0.488	0.741(0.319-1.719)	0.485
	CG+GG	384(97.5)	381(96.7)		1.000			1.000	
rs2632516	Genotype								
	GG	114(29.1)	104(26.9)	0.946	1.223(0.815-1.833)	0.331	0.672	1.189(0.786-1.797)	0.413
	GC	200(51.0)	195(50.5)		1.144(0.795-1.646)	0.469	0.429	1.132(0.781-1.641)	0.513
	CC	78(19.9)	87(22.5)		1.000			1.000	
	Allele								
	G	428(54.6)	403(52.2)	0.893	1.101(0.902-1.344)	0.345			
	C	356(45.4)	369(47.8)		1.000				
	Dominant model								
	GG+GC	314(80.1)	299(77.5)	0.811	1.171(0.830-1.653)	0.368	0.623	1.152(0.811-1.637)	0.430
	CC	78(19.9)	87(22.5)		1.000			1.000	
	Recessive model								
	GG	114(29.1)	104(26.9)	0.441	1.112(0.813-1.521)	0.507	0.274	1.089(0.791-1.500)	0.601
	GC+CC	278(70.9)	282(73.1)		1.000			1.000	
rs2877877	Genotype								
	GG	31(7.9)	46(12.0)	6.889	0.513(0.312-0.845)	0.009	7.006	0.502(0.301-0.836)	0.008
	GA	142(36.3)	171(44.6)	8.939	0.632(0.468-0.854)	0.003	9.622	0.615(0.452-0.836)	0.002
	AA	218(55.8)	166(43.3)		1.000			1.000	
	Allele								
	G	204(26.1)	263(34.3)	12.434	0.675(0.543-0.840)	<0.001			
	A	578(73.9)	503(65.7)		1.000				
	Dominant model								
	GG+GA	173(44.2)	217(56.7)	11.863	0.607(0.457-0.806)	0.001	12.617	0.591(0.442-0.790)	<0.001

	AA	218(55.8)	166(43.3)		1.000			1.000	
	Recessive model								
	GG	31(7.9)	46(12.0)	3.552	0.631(0.391-1.019)	0.059	3.544	0.624(0.382-1.020)	0.060
	GA+AA	360(92.1)	337(88.0)		1.000			1.000	
rs2067079	Genotype								
	TT	31(7.9)	45(11.3)	11.981	0.400(0.238-0.672)	0.001	11.870	0.397(0.235-0.672)	0.001
	TC	187(47.7)	251(63.2)	28.131	0.432(0.317-0.589)	<0.001	30.059	0.412(0.300-0.565)	<0.001
	CC	174(44.4)	101(25.4)		1.000			1.000	
	Allele								
	T	249(31.8)	341(42.9)	20.968	0.618(0.503-0.760)	<0.001			
	C	535(68.2)	453(57.1)		1.000				
	Dominant model								
	TT+TC	218(55.6)	296(74.6)	30.582	0.482(0.316-0.578)	<0.001	32.215	0.409(0.301-0.557)	<0.001
	CC	174(44.4)	101(25.4)		1.000			1.000	
	Recessive model								
	TT	31(7.9)	45(11.3)	2.635	0.672(0.415-1.086)	0.105	2.261	0.690(0.425-1.119)	0.133
	TC+CC	361(92.1)	352(88.7)		1.000			1.000	

Adjusted by gender and age; SLE: systemic lupus erythematosus; HC: health controls

Table S9 Primers sequences used for qRT-PCR

Gene	Forward	Reverse
Linc0597	F:5' TTGGATTCATCCCGTTCACCTCCA 3'	R:5' CAGCATGACGATCAAGCGAGATTC 3'
Linc0949	F:5' GCGAAGAGACCACCAAACAG 3'	R:5' AAAGAAGCAGGACTACCCACT 3'
Lnc-DC	F:5' GATCGTCATCCCTTCCTGG 3'	R:5' GAAACAACCCCTCTTCCCTG 3'
GAS5	F:5' TATGGTGCTGGGTGCGGAT 3'	R:5' CCAATGGCTTGAGTTAGGCTT 3'
β-actin	F: 5' CACGAAACTACCTTCAACTCC 3'	R: 5' CATACTCCTGCTTGCTGATC 3'

SNPs in linc0597 (BZRAP1-AS1) and its promoter region

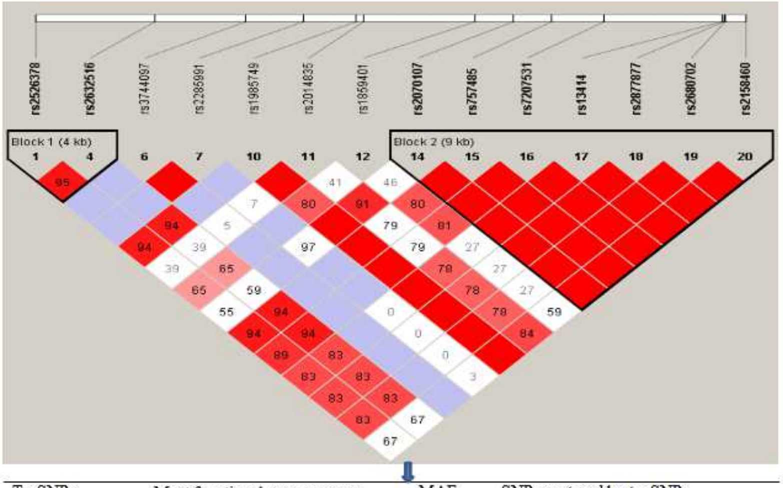

Information of SNPs

TagSNPs	Most functional consequence	MAF	SNPs captured by tagSNPs
rs7207531	Intron variant	0.45	rs1985749(intron variant)<br>rs757485(intron variant)
rs2877877	3 prime UTR variant	0.18	rs2680702(3 prime UTR variant)<br>rs13414(3 prime UTR variant)
rs2632516	Exon variant	0.47	rs2526378(Intron variant)
rs2070107	3 prime UTR variant	0.13	-
rs2014835	Intron variant	0.12	-
rs3744097	Intron variant	0.04	-
rs1859401	Intron variant	0.42	-
rs2158460	Intron variant	0.16	-
rs2285991	Exon variant	0.04	-

Filter out: intron variant and MAF<0.05

Finally included：rs2070107,rs2632516,rs2877877

Figure S1

SNPs in lnc-DC and its promoter region

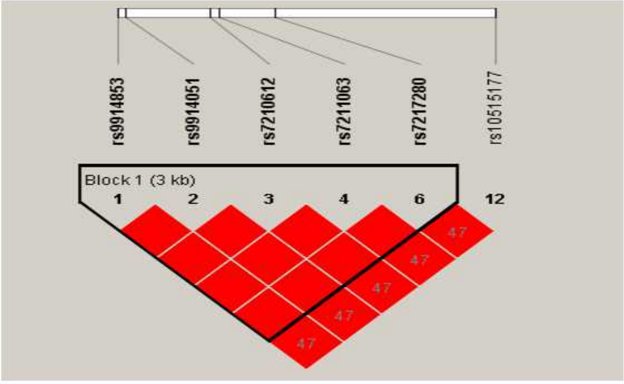

Information of SNPs

TagSNPs	Most functional consequence	MAF	SNPs captured by TagSNPs
rs10515177	Upstream variant 2KB	0.2	-
rs7210612	Intron variant	0.1	rs9914853(Intron variant)
			rs7217280(Intron variant)
			rs9914051(Intron variant)
			rs7211063(Intron variant)

Filter out: intron variant and MAF<0.05

Finally included : rs10515177

Figure S2

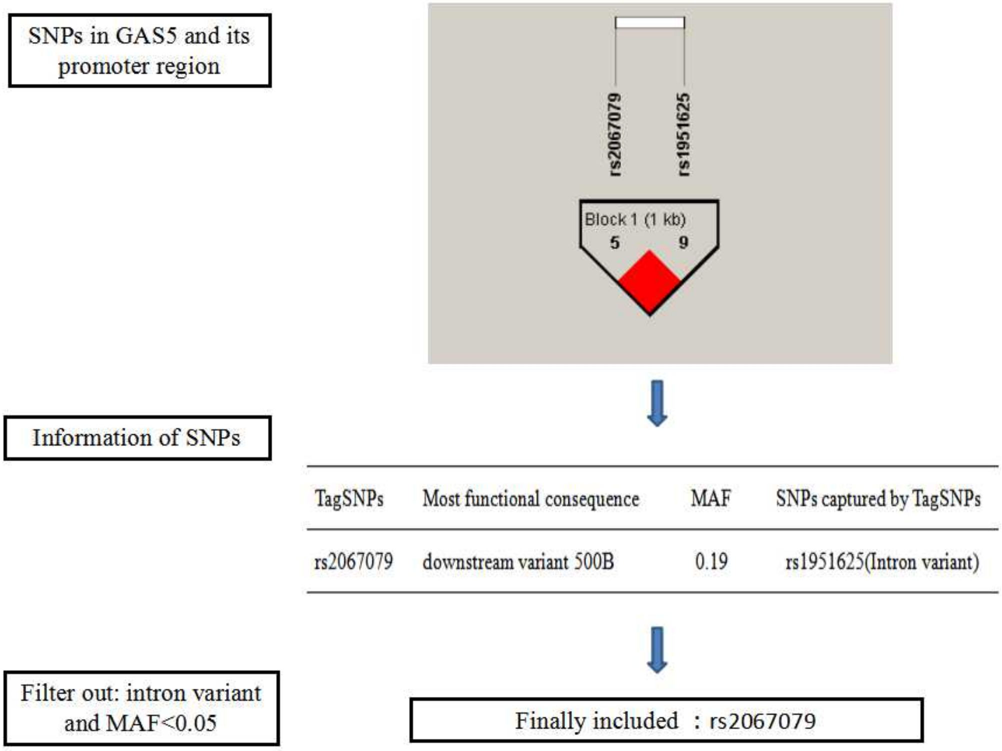

Figure S3

Figure legend

Figure S1 Overall flow of SNP selection and relative information of the tagSNPs in linc0597 (BZRAP1-AS1)

Figure S2 Overall flow of SNP selection and relative information of the tagSNPs in lnc-DC

Figure S3 Overall flow of SNP selection and relative information of the tagSNPs in GAS5

MAF: minor allele frequency
